# Quality Differences Among Fish- and Algae-Derived Omega-3 Supplements: Insights from Physicochemical, Oxidative, and Thermal Analyses

**DOI:** 10.3390/foods15213786

**Published:** 2026-10-22

**Authors:** Iga Piasecka-Lenartowicz, Klara Golec, Marcelina Lis, Marta Siol, Magdalena Wirkowska-Wojdyła, Ewa Ostrowska-Ligęza, Agata Górska

**Affiliations:** 1 Department of Chemistry, Faculty of Bioeconomy and Food Systems, Warsaw University of Life Sciences, 159 Nowourysynowska Street, 02-776 Warsaw, Poland; marta_siol@sggw.edu.pl (M.S.); magdalena_wirkowska@sggw.edu.pl (M.W.-W.); ewa_ostrowska-ligeza@sggw.edu.pl (E.O.-L.); 2 Faculty of Biology and Biotechnology, Warsaw University of Life Sciences, 159 Nowourysynowska Street, 02-776 Warsaw, Polands227102@sggw.edu.pl (M.L.)

**Keywords:** omega-3, dietary supplements, algae, fish oil

## Abstract

Omega-3 long-chain polyunsaturated fatty acids, particularly eicosapentaenoic acid (EPA) and docosahexaenoic acid (DHA), are widely consumed due to their recognized health benefits. Consequently, dietary supplements containing fish oil or algae-derived oils have become increasingly popular. However, the high susceptibility of polyunsaturated fatty acids to oxidation raises concerns regarding the quality and stability of such products. This study aimed to comprehensively evaluate the physicochemical quality, oxidative stability, thermal behavior, and fatty acid composition of omega-3 dietary supplements available on the Polish over-the-counter market, with particular emphasis on differences between fish- and algae-derived products. Twelve supplements were analyzed, including eight fish oil-based and four algae-derived formulations. Fatty acid composition was determined by gas chromatography (GC), oxidative stability by pressure differential scanning calorimetry (PDSC), and melting characteristics by differential scanning calorimetry (DSC). In addition, acid value (AV), peroxide value (PV), p-anisidine value (p-AnV), and total oxidation index (TOTOX) were measured. Fish oil supplements contained both EPA (17.50–38.76%) and DHA (13.42–36.83%), whereas algae-derived products were characterized by high DHA content (36.83–62.79%) and low EPA levels. Oxidative stability varied markedly among samples, with oxidation induction times ranging from 6.73 to 18.71 min. TOTOX values ranged from 11.57 to 36.43, indicating substantial differences in oxidative quality. Thermal analysis further revealed distinct melting behaviors associated with lipid composition. This study provides a comprehensive comparison of fish- and algae-based omega-3 supplements, integrating compositional, oxidative, and thermal analyses with technological factors such as packaging and antioxidant systems. The results highlight considerable variability in the quality of commercially available products and emphasize the importance of multidimensional analytical approaches for reliable evaluation of omega-3 supplements.

## 1. Introduction

Omega-3 fatty acids are essential unsaturated fatty acids that play a crucial role in human health. Among them, the most important omega-3 fatty acids are eicosapentaenoic acid (EPA, C20:5 n-3) and docosahexaenoic acid (DHA, C22:6 n-3). Because the human body has a limited capacity to synthesize these long-chain omega-3 fatty acids, an adequate intake is necessary. The primary natural sources of omega-3 fatty acids include mainly fatty sea fish and sea algae. In addition to dietary intake, these fatty acids can also be provided through dietary supplements, most commonly in the form of encapsulated oils [1]. A review article by Calder et al. [2] compares dietary recommendations regarding omega-3 intake worldwide. In 2010, the European Food Safety Authority (EFSA) recommended EPA and DHA adequate intake (AI) at 250 mg/day for adults. The Food and Agriculture Organization of the United Nations (FAO) states that 250 mg—two grams of EPA and DHA—is an acceptable macronutrient distribution range. The recommendations may differ among countries or organizations; however, the most common one is 250 mg/day intake of DHA and EPA. Higher doses are recommended for women during pregnancy or lactation and as cardiovascular disease prevention.

Omega-3 fatty acids have a wide range of implications for human health. Cardiovascular diseases are among the most common diseases in humans. EPA and DHA affect the circulatory system through their anti-inflammatory effects and protect against oxidative stress [3,4,5]. They play an important role in regulating blood pressure, peripheral blood vessels, and coagulation, among other things [6]. DHA reduces the risk of atrial fibrillation. On the contrary, EPA is important in lowering the coronary artery disease risk [7]. In terms of higher omega-3 intake by pregnant women, studies indicate significant benefits for fetal brain and retinal development, which may lead to development of the fetus [8,9]. Some studies also indicate the impact of EPA and DHA on the prevention and cognitive functions of Alzheimer’s disease [10,11].

It is important to note that with many dietary supplements on the market, the quality of these products can vary significantly, depending on the product’s origin, composition, manufacturer, packaging, exposure to light and oxygen, etc. [12]. Some adulterations and deviations have been previously reported [13,14,15]. The quality of omega-3 dietary supplements can be evaluated using a range of physicochemical parameters that reflect both their compositional characteristics and oxidative status [16]. Lipid quality is commonly assessed through indicators of hydrolytic and oxidative degradation [17]. The acid value (AV) provides information about the degree of lipid hydrolysis and the presence of free fatty acids formed as a result of triglyceride breakdown. The peroxide value (PV) reflects the concentration of primary oxidation products, mainly lipid hydroperoxides, which are formed during the initial stages of lipid oxidation. The p-anisidine value (p-AnV), in turn, indicates the level of secondary oxidation products such as, for example, aldehydes that are generated during the decomposition of hydroperoxides. A more comprehensive indicator of lipid oxidation is the total oxidation index (TOTOX), which combines both primary and secondary oxidation parameters. In addition to oxidation markers, fatty acid composition is an important characteristic determining the nutritional value and technological properties of omega-3 supplements. Therefore, the fatty acid profile is commonly analyzed using gas chromatography (GC), which allows precise identification and quantification of individual fatty acids, including the key omega-3 polyunsaturated fatty acids such as EPA and docosahexaenoic acid DHA. Furthermore, the stability of oils rich in polyunsaturated fatty acids can be assessed using pressure differential scanning calorimetry (PDSC), which enables determination of oxidation induction time under controlled oxidative conditions. Thermal properties of the lipid fraction, including melting behavior and phase transitions, may also be evaluated using differential scanning calorimetry (DSC), providing insight into the structural organization of the lipid matrix and the presence of different triacylglycerol fractions. In addition to chemical and thermal analyses, technological factors such as packaging type, antioxidant additives, and capsule coating composition may influence the stability and overall quality of dietary supplements.

The aim of the present study was to evaluate the quality of omega-3 dietary supplements available on the Polish over-the-counter (OTC) market, including a comparison between products derived from fish oil and algae-based sources. The analysis included determination of acid value, peroxide value, p-anisidine value, and TOTOX index, as well as fatty acid composition determined by gas chromatography. Additionally, oxidative stability was assessed using PDSC, while melting characteristics were analyzed by DSC. Furthermore, statistical analyses were performed to examine associations between oil source (fish or algae), packaging type, and the physicochemical properties and stability of the supplements.

## 2. Materials and Methods

### 2.1. Materials

The 12 dietary supplements—four vegan with algae-origin omega-3 (1V–4V) and eight formulated with fish oil-derived omega-3 (1F–8F)—were analyzed. All the dietary supplements were purchased from a pharmacy in Poland. They differed in terms of brand, country of production, packaging, antioxidant ingredients, capsule coating and declared EPA/DHA percentages (Table 1).

### 2.2. Comprehensive Analysis

#### 2.2.1. Fatty Acid Profile

The fatty acid profile was determined by gas chromatography (GC). Prior to analysis, oil samples were converted to fatty acid methyl esters (FAMEs) by transesterification with methanolic potassium hydroxide, in accordance with PN-EN ISO 5509:2001 [18]. The resulting FAMEs were analyzed using a YL6100 gas chromatograph (Young Lin Instrument Co., Ltd., Anyang, Republic of Korea) equipped with a flame ionization detector (FID) and a 60 m BPX70 capillary column (SGE Analytical Science, Milton Keynes, UK). Analyses were performed in split injection mode (split ratio 1:50), with nitrogen as the carrier gas. The injector and detector temperatures were set at 225 °C and 250 °C, respectively. The oven temperature program was as follows: initial temperature of 70 °C (held for 0.5 min), increased to 160 °C at 15 °C/min, then raised to 200 °C at 1.1 °C/min and held for 6 min, followed by a final increase to 225 °C at 30 °C/min. Fatty acids were identified by comparing their retention times with those of a FAME standard mixture (Supelco 37 Component FAME Mix, Sigma-Aldrich, Bellefonte, PA, USA). The relative content of each fatty acid was expressed as a percentage of the total identified fatty acids in the sample [19].

#### 2.2.2. Oxidative Stability

The oxidative stability index—oxidation induction time (OIT)—was determined using pressure differential scanning calorimetry (PDSC). Samples (3–4 mg) were placed in open aluminum pans, while an empty pan served as the reference. Measurements were performed using a DSC Q20 instrument (TA Instruments, New Castle, DE, USA). The analyses were conducted under isothermal conditions at 120 °C, with an initial oxygen pressure of 1400 kPa. The OIT (min) was determined from the heat flow–time curve using TA software (v. 4.5A) [20].

#### 2.2.3. Melting Characteristics

Melting characteristics were evaluated using differential scanning calorimetry (DSC) with a DSC Q200 calorimeter (TA Instruments, New Castle, DE, USA). Samples (3–4 mg) were weighed into aluminum pans, hermetically sealed with lids, and analyzed against an empty reference pan. Measurements were conducted under a nitrogen atmosphere. The analysis protocol began with heating the samples to 80 °C to completely melt crystalline structures and eliminate thermal memory. Subsequently, the samples were cooled to −80 °C and reheated to 80 °C at a scanning rate of 15 °C/min [21]. The heat flow-temperature curve was analyzed in TA software to assess the melting onset and peak temperatures, as well as the enthalpy of the event.

#### 2.2.4. Acid Value, Peroxide Value and TOTOX

The acid value (AV), used as an indicator of the degree of oil hydrolysis, was determined according to the AOCS official method Cd 3d—63 [22]. The peroxide value (PV), reflecting the content of primary oxidation products, was measured in accordance with AOCS method Cd 8—53 [23]. The p-anisidine value (p-AnV), representing the level of secondary oxidation products, was determined following AOCS method Cd 18—90 [24]. The total oxidation (TOTOX) index, expressing the overall oxidative status of the oil, was calculated according to Equation (1).

TOTOX = (2 × PV) + p-AnV(1)

#### 2.2.5. Statistical Analysis

All analyses were performed in triplicate, and the results are expressed as mean ± standard deviation (SD). The results were analyzed using Statistica 13.3 software (TIBCO, Palo Alto, CA, USA) using analysis of variance (ANOVA) followed by post hoc Tukey’s test. The significance level was *p* < 0.05. Additionally, the analysis of correlations between some specific features of supplements with their chemical characteristics was performed, PCA was carried out, and results were also shown as a heatmap. An important limitation of this study is the uneven distribution of packaging types across the two oil-source categories. While fish-oil supplements were available in cardboard, plastic, and glass packaging, algae-based supplements were available only in cardboard and plastic packaging, with plastic containers predominating in this group.

## 3. Results and Discussion

### 3.1. The Fatty Acid Profile

The complete results of the gas chromatographic analysis are provided in Supplementary Material Table S1. The gas chromatography analysis confirmed clear compositional differences between fish oil- and algae-derived supplements. Since the method did not reflect the content of particular fatty acids, a comparison with the label is not possible. Fish oil supplements were characterized by a relatively balanced contribution of EPA (C20:5 n-3) and DHA (C22:6 n-3), sharing 17.50–38.76% and 13.42–24.73% of total FAMEs, respectively. Those results are consistent with the natural fatty acid pattern of marine fish oils rich in long-chain n-3 PUFA [25,26]. Fish oil supplements also exhibited variable levels of oleic acid (C18:1 n-9), 5.15–22.37%, suggesting differences in refining degree or blending practices but still being consistent with previous findings [25,26]. Samples with higher monounsaturated fatty acid (MUFA) content may demonstrate slightly improved oxidative stability compared to samples with a predominance of PUFA [27].

Among algae-derived supplements, DHA was the predominant fatty acid, sharing 36.83–62.79%, reflecting the metabolic profile of *Schizochytrium* sp., a well-known industrial source of DHA [28,29]. Except for the 1V sample (18.26% EPA), the algae oil did not exhibit a high percentage share of EPA (0.64–3.39% EPA). This may suggest technological blending or strain-specific production differences. Also, the studies claim that different conditions of algae culture may influence the synthesis of fatty acids, e.g., substrate composition and availability, as well as genetic modifications [30,31,32]. From a nutritional standpoint, such variation may influence the intended physiological effect, as EPA and DHA exhibit partially distinct biological activities [33]. The presence of lignoceric acid (C24:0) at relatively high levels (12.80–14.01%) in 2V–4V indicates either residual algal lipid fractions or the addition of structured lipid components. The longer the chain of the saturated fatty acid, the higher melting point they can reach [34]. Lower percentage share of PUFAs and higher percentage of SFAs also improve oxidative stability [35], which was partially reflected in PDSC results—samples 3V and 4V had significantly longer oxidation induction times.

**Figure 1:**
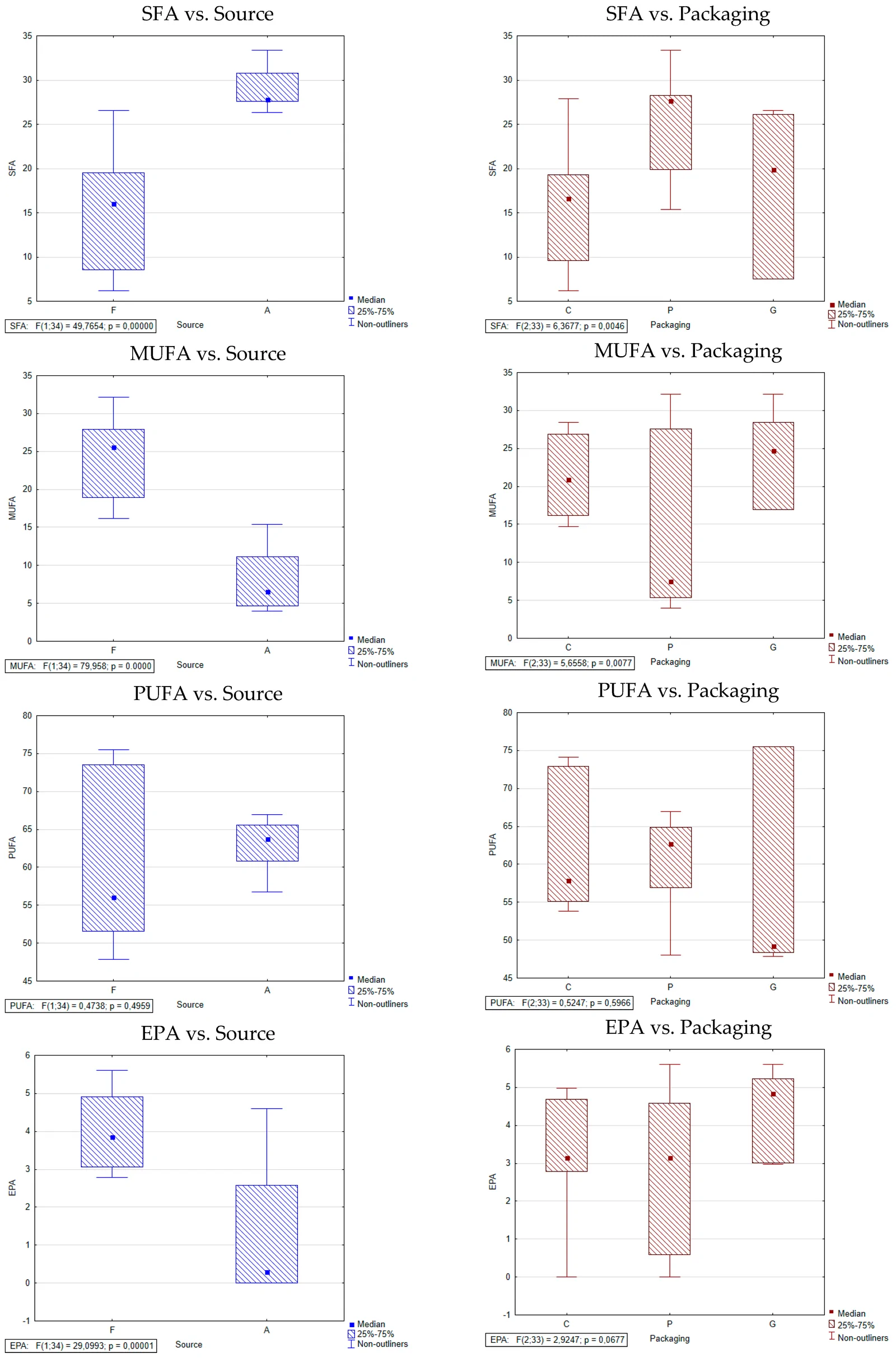

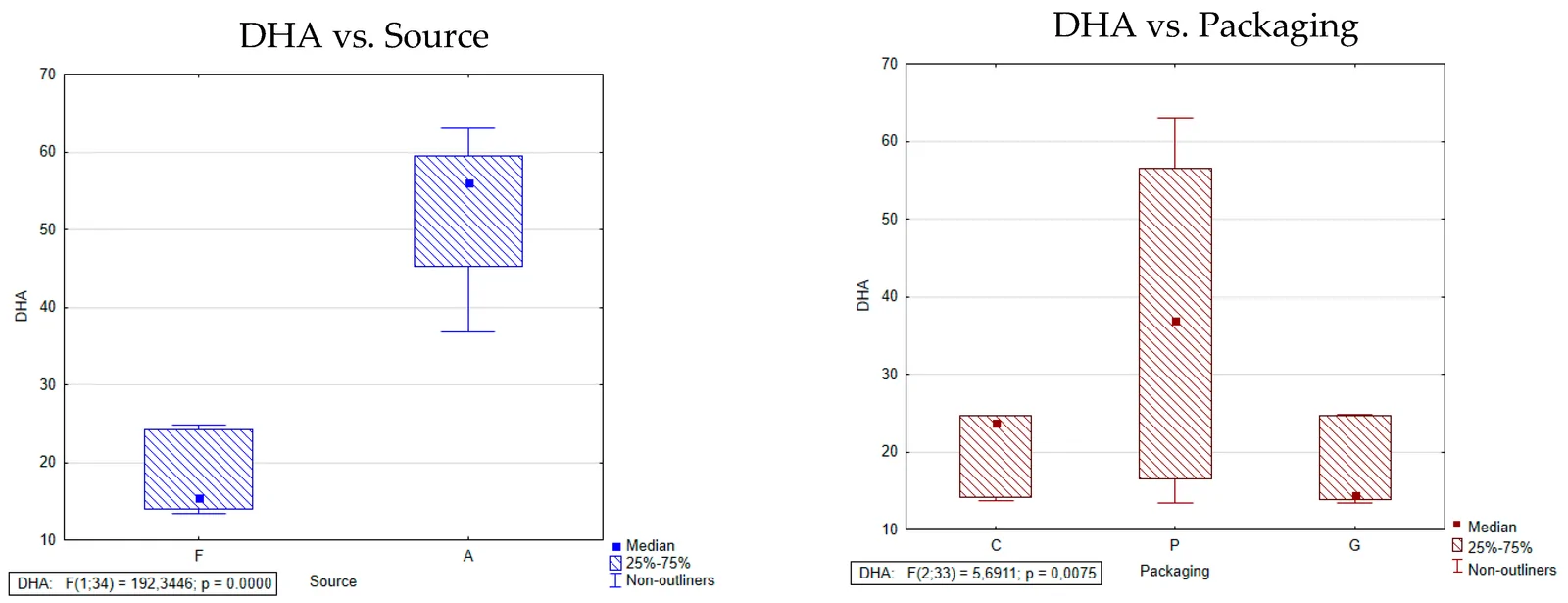
The biplots showing relationships between the sum of SFA, MUFA, PUFA percentage share, EPA and DHA percentage share, and source of omega-3 dietary supplement (F—fish or A—algae) and packaging (C—cardboard, P—plastic, G—glass).

The influences of the source of the supplement and packaging on the sum of SFA, MUFA and PUFA, as well as the percentage share of EPA and DHA, were studied, and the results are presented on biplots (Figure 1). The sum of SFA and MUFA percentages were significantly influenced by the source of the dietary supplement. The association with packaging category was also observed. The source of omega-3 supplement had a significant influence on the percentage of DHA and EPA in tested samples. Interestingly, the packaging type was important only in the case of DHA percentage share but did not influence the EPA percentage share.

### 3.2. Oxidation Induction Time

The results of the PDSC analysis are presented in Table 2. Among fish oil supplements, 8F demonstrated the highest oxidative stability (18.71 min), which may be attributed to its antioxidant formulation and packaging in orange glass, limiting light exposure. In contrast, 7F showed the lowest stability among fish oils (7.09 min), despite containing vitamin E as an antioxidant. This suggests that antioxidant type, concentration, and synergistic effects (e.g., rosemary extract in 6F) may be more decisive than the mere presence of tocopherols. The oxidative stability using PDSC has not been widely studied. Araújo et al. [36] tested OIT of refined fish oil at a lower temperature of 100 °C. The OIT of the sample was 53 min. Algae-derived supplements generally exhibit lower OIT values (6.73–9.35 min) compared with fish oils. The higher DHA proportion in these oils may partly explain this observation, as DHA (six double bonds) is more susceptible to peroxidation than EPA (five double bonds). However, 3V and 4V demonstrated moderate stability (approximately 9 min), suggesting effective antioxidant protection. The formulation and concentration of antioxidant agent is crucial in terms of oxidative resistance. Unfortunately, the exact amount of added antioxidant is unknown. It can, however, be noted that in fish-derived samples, vitamin E or tocopherol mixture are the most common antioxidants, except for sample 6F, where rosemary extract was added as well. In algae-derived samples, tocopherol mixture was combined with ascorbic acid to improve oxidative stability even more. Shen et al. [37] discovered that addition of an optimized mixture of antioxidants, including ascorbyl pamitate, vitamin E, phytic acid and tea polyphenols, to algae oil resulted in 3.8-fold prolonged shelf life of the tested samples.

**Figure 2:**
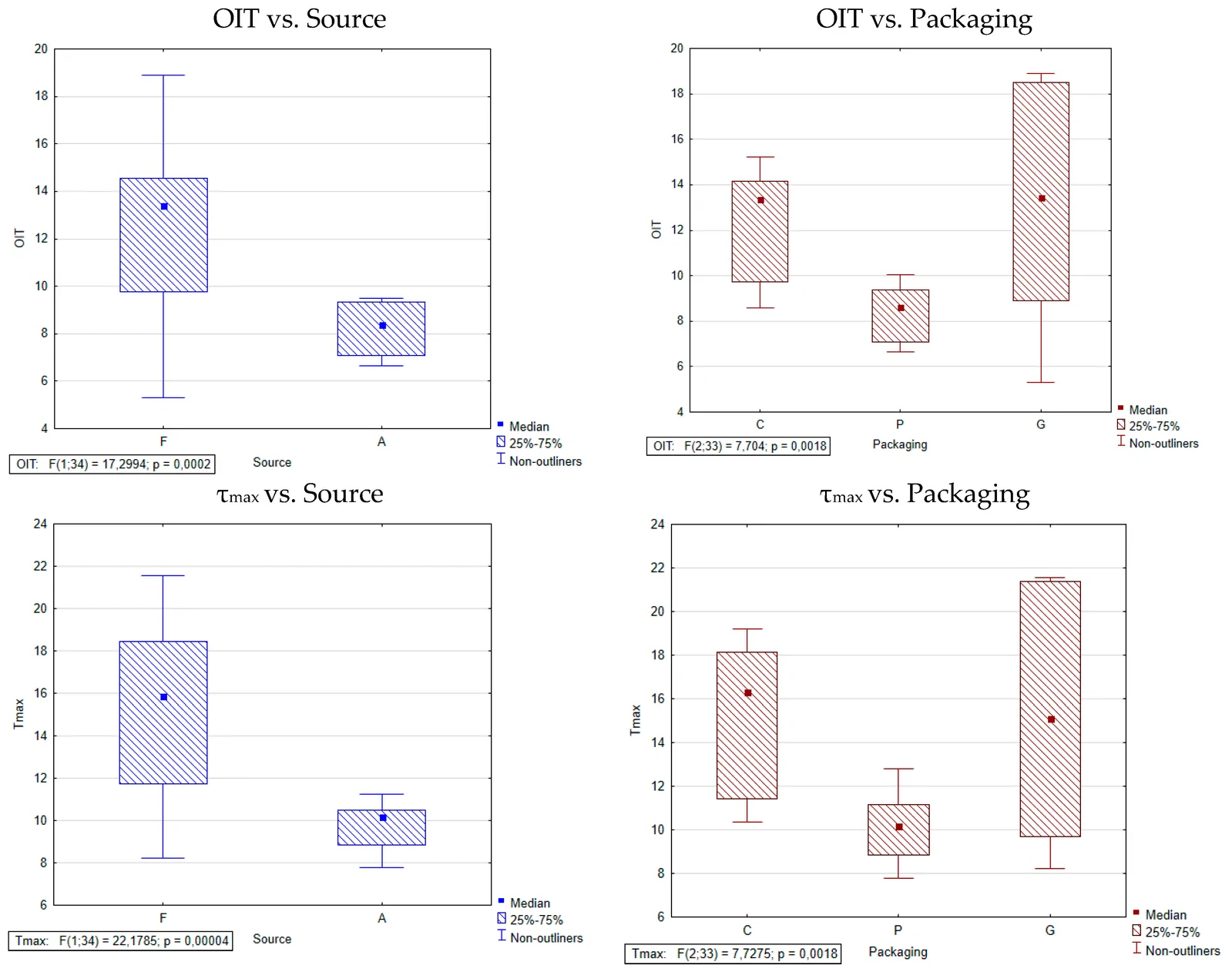
Biplots showing relationships between OIT and τmax and the source of omega-3 dietary supplement (F—fish or A—algae) and packaging (C—cardboard, P—plastic, G—glass).

Based on biplots (Figure 2) and statistical analysis, oxidative stability varied across packaging categories. However, these differences cannot be attributed solely to packaging because packaging type and oil source were unevenly distributed among the analyzed products. Supplements stored in orange glass tended to show better stability than those packed in cardboard boxes and plastic containers. In terms of the source of oil, fish-derived supplements were characterized by significantly longer OIT and τmax values. These findings underline the technological importance of oxygen, light protection, and antioxidant strategy in omega-3 supplement formulation [13]. The PDSC results were also consistent with the chemical quality parameters of the omega-3 supplements, including AV, PV, and TOTOX values, as discussed in Section 3.4.

### 3.3. Thermal Behavior

**Figure 3:**
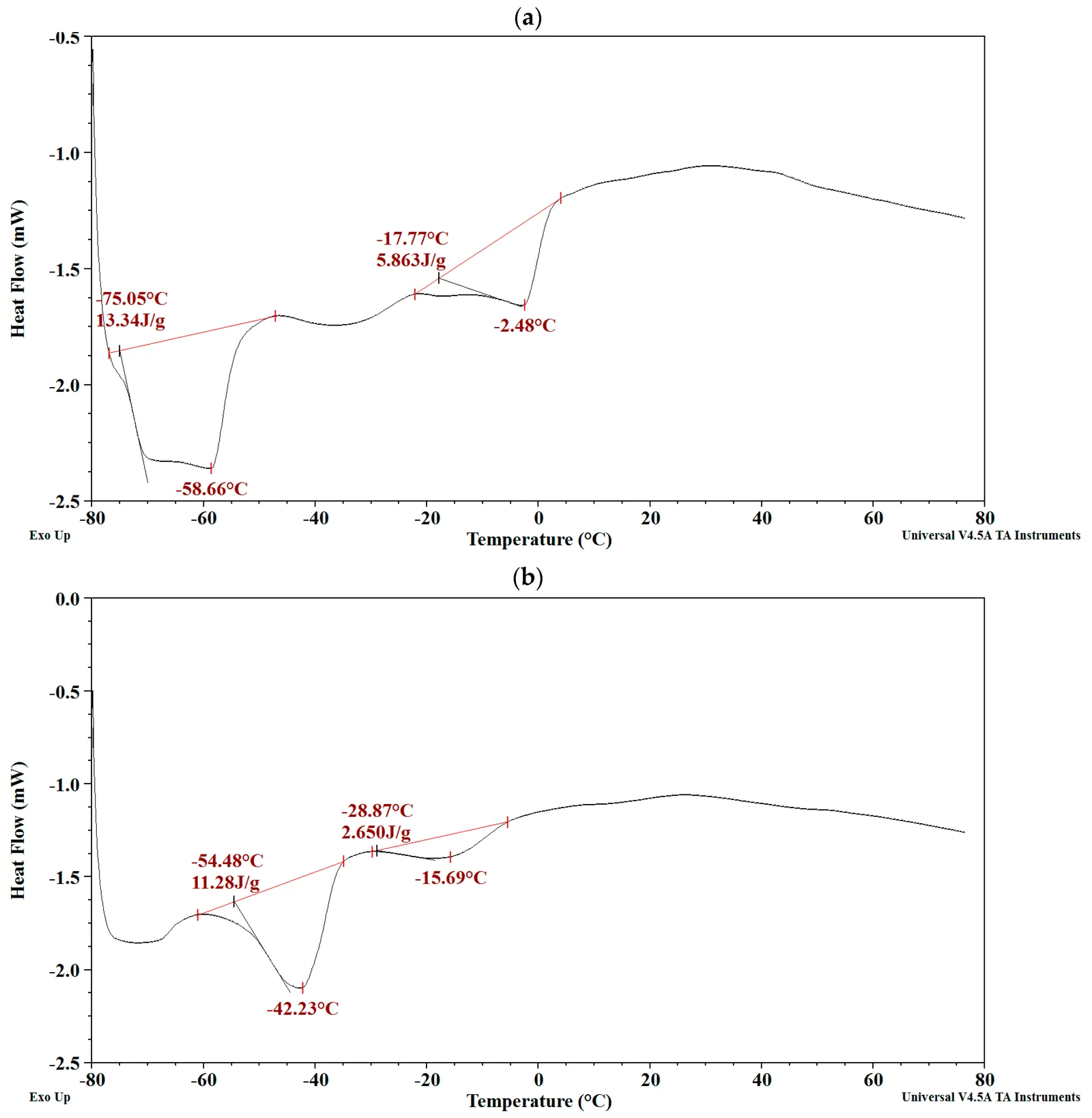
Example of DSC curve of fish omega-3 sample 8F (**a**); example of DSC curve of algae omega-3 sample 3V (**b**).

The results obtained in the DSC study are presented in Table 3, and the examples of obtained DSC curves are shown in Figure 3. This study revealed two main endothermic transitions in most samples, corresponding to melting of low-melting PUFA-rich fractions (T_on1_, T_max1_) and higher-melting monounsaturated and saturated lipid fractions (T_on2_, T_max2_). Fish oils generally exhibited lower onset temperatures for the first transition (down to −74.65 °C), reflecting high unsaturation levels. Samples such as 6F, 7F, and 8F showed particularly low Ton1 values, consistent with elevated long-chain PUFA content. Algae oils displayed first melting transitions at slightly higher temperature (around −54 °C), which may be linked to their fatty acid distribution and higher content of very-long-chain saturated fatty acids (e.g., C24:0). The second melting transition in algae oils occurred at markedly lower temperatures (−28 to −17 °C) compared with fish oils (approximately −21 to −2 °C), suggesting differences in triacylglycerol structure and crystallization patterns. Fish oils were previously described as ones with an onset point of transition in low temperatures, even down to −90 °C [38,39]. Melting behavior of algae oil was not studied widely. Wang et al. [40] recorded DSC for algae-enriched structured lipids, and their onset temperatures of first melting were in a range of −46 to −19 °C. The melting points of saturated fatty acids rise as their chain length increases, likely because intermolecular dispersion forces become stronger with a greater number of carbon atoms in the chain. In contrast, for unsaturated fatty acids, a higher number of double bonds leads to a lower melting point [41].

Enthalpy values (ΔH) varied substantially among samples, indicating differences in crystalline fraction proportion and lipid organization. The enthalpy values are consistent with those previously reported for fish oil [39]. Samples with higher ΔH_2_ (e.g., 4F, 5F, 4V) likely contained greater proportions of higher-melting structured fractions, which may influence capsule stability. The observed thermal diversity confirms that even supplements with similar declared EPA/DHA levels may differ in melting characteristics due to raw material origin and processing history.

### 3.4. Chemical Characteristics

**Figure 4:**
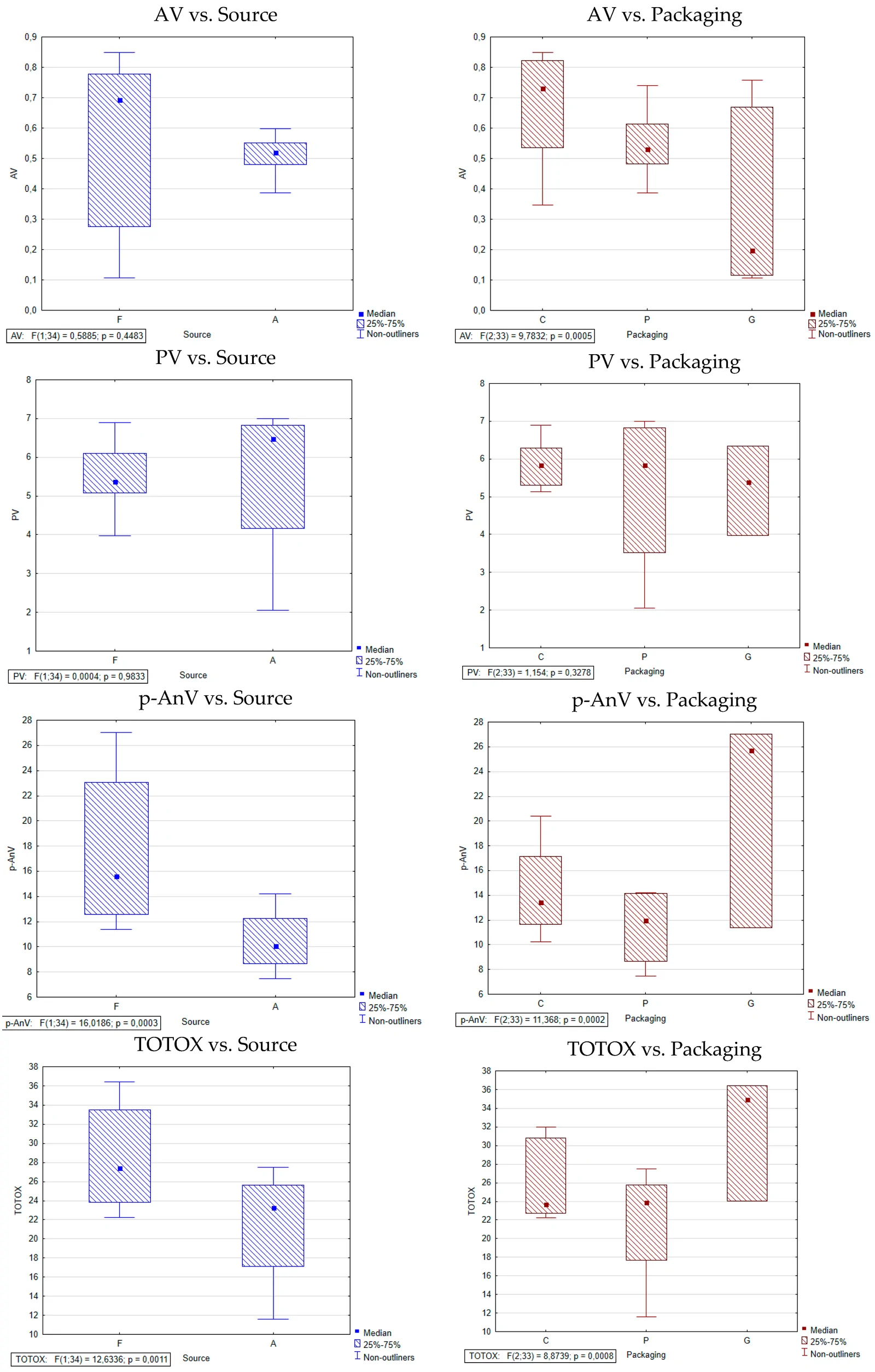
The biplots showing relationships between AV, PV, p-AnV, TOTOX, and the source of omega-3 dietary supplement (F—fish or A—algae) and packaging (C—cardboard, P—plastic, G—glass).

Besides the stability of omega-3 dietary supplements measured using PDSC and DSC, the chemical characteristics of oil quality, i.e., acid value, peroxide value, and p-anisidine value with counted TOTOX, were studied and are summarized in Table 4. The biplots regarding statistical approach of obtained results are shown in Figure 4. Acid values (0.11–0.83 mg KOH/g) were generally low, indicating limited hydrolytic degradation and appropriate storage conditions. The values of AV were below threshold values claimed by Codex Alimentarius (≤3 mg KOH/g) [42]. The lowest AV values (6F, 8F) suggest good refining efficiency and minimal lipolytic activity. Peroxide values ranged from 2.08 to 6.97 meq O_2_/kg. However, the Global Organization for EPA and DHA Omega-3 (GOED) as well as Codex Alimentarius recommend PV to remain below 5 meq O_2_/kg [42,43], so several samples exceeded the recommended limit. The lowest PV was noted for 2V (2.08 meq O_2_/kg), reflecting excellent primary oxidation control. Based on previous findings, the capsule form of fish oil is the most efficient delivery method in terms of oxidation prevention, compared to syrup form and chewable form [44]. In the cited study, chemical characteristics during storage were evaluated, and the maximum values of PV, p-AnV and TOTOX reached by samples were 7.62, 19.58, and 30.44 meq/kg oil, respectively. Kolanowski [45] studied different fish oils and confirmed that gelatin capsules did not fully prevent oxidative deterioration and that stability was strongly influenced by formulation factors, including the type and effectiveness of antioxidants, as well as the initial quality of the oil. The presence of oxidation products may be harmful to consumers; thus, the control of chemical properties of fish and algae oil is a crucial part of manufacturing [46]. However, secondary oxidation products (p-AnV) showed wider variation (7.46–27.03). Samples 6F and 7F exhibited particularly high p-AnV values (>25), resulting in the highest TOTOX indices (36.43 and 34.96, respectively). This indicated progression from primary to secondary oxidation stages. In contrast, 2V showed the lowest TOTOX value (11.57), reflecting superior overall oxidative quality. The GOED monograph [43] recommended p-AnV and TOTOX values to be below 20 and 26, respectively. It can be concluded that three samples (3F, 6F and 7F) exceeded p-AnV and five samples (3F, 4F, 6F, 7F, 1V) exceeded TOTOX limits.

Numerous studies have investigated the quality characteristics of fish-oil delivered omega-3 dietary supplements. A study of the French market showed that all tested supplements complied with GOED recommendations in terms of PV, p-AnV and TOTOX [47]. The US-market study [13] of 48 dietary supplements revealed that they complied with 85% of recommended PV and with 96% and 87% of recommended p-AnV and TOTOX, respectively. For the purpose of p-AnV and TOTOX assessments, only unflavored oils were taken into account. They also claimed that improvement of oxidative quality may be achieved through optimization of processing conditions, minimization of oxygen exposure during encapsulation, and the use of appropriate antioxidant combinations tailored to expected storage conditions. The study by Hands et al. [48] evaluated 72 marine and microalgal oil omega-3 supplements sold in the USA. A high proportion of described omega-3 supplements exceeded recommended oxidation limits, with flavored products showing markedly higher non-compliance (up to 68% for TOTOX and 62% for p-AnV) compared to unflavored ones. These findings indicate that flavoring agents can significantly interfere with oxidation assessment, particularly p-AnV, potentially leading to overestimation of secondary oxidation in such products.

Interestingly, high OIT did not always correspond with low TOTOX values. For example, sample 8F exhibited the highest OIT (18.71 min) but moderate TOTOX (24.06), suggesting that induction time under accelerated conditions does not directly mirror accumulated oxidation products formed during storage. Based on the biplots shown in Figure 4, the p-AnV and TOTOX were dependent on the source of the omega-3 supplement, and algae-derived capsules resulted in lower numbers for both parameters. The results of AV, p-AnV and TOTOX were also dependent on the packaging type. For AV, orange glass packaging was favorable, and for p-AnV and TOTOX, plastic packaging resulted in lower values. However, these patterns should be interpreted cautiously, as the independent contribution of packaging could not be distinguished from the effect of oil source and other product-specific factors, especially in the case of vegan supplements.

### 3.5. Statistical Approach

**Figure 5:**
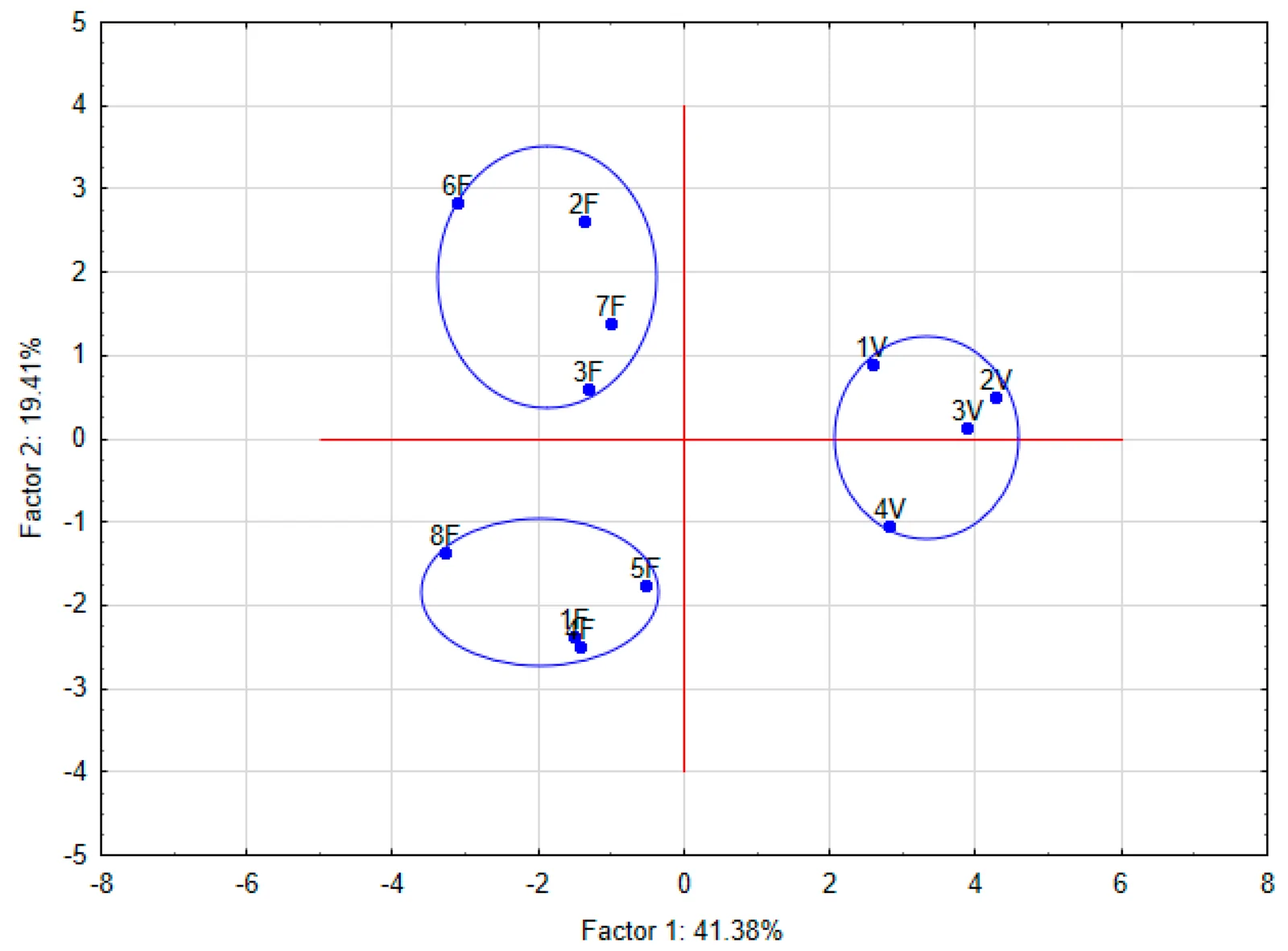
Principal component analysis (PCA) of the omega-3 dietary supplements.

Multivariate statistical analysis provided clear differentiation among the analyzed omega-3 supplements. Principal component analysis (PCA) showed that the first two principal components explained a substantial proportion of the total variance, with PC1 accounting for 41.38% and PC2 for 19.41% (Figure 5). A distinct separation between fish oil (F) and algae-derived (V) supplements was observed along PC1, which was consistent with the differences in fatty acid composition observed between these two groups. Algae-based samples (1V–4V) formed a compact cluster on the positive side of PC1, reflecting their more uniform composition characterized by high DHA content, whereas fish oil samples were distributed on the negative side, exhibiting greater variability.

**Figure 6:**
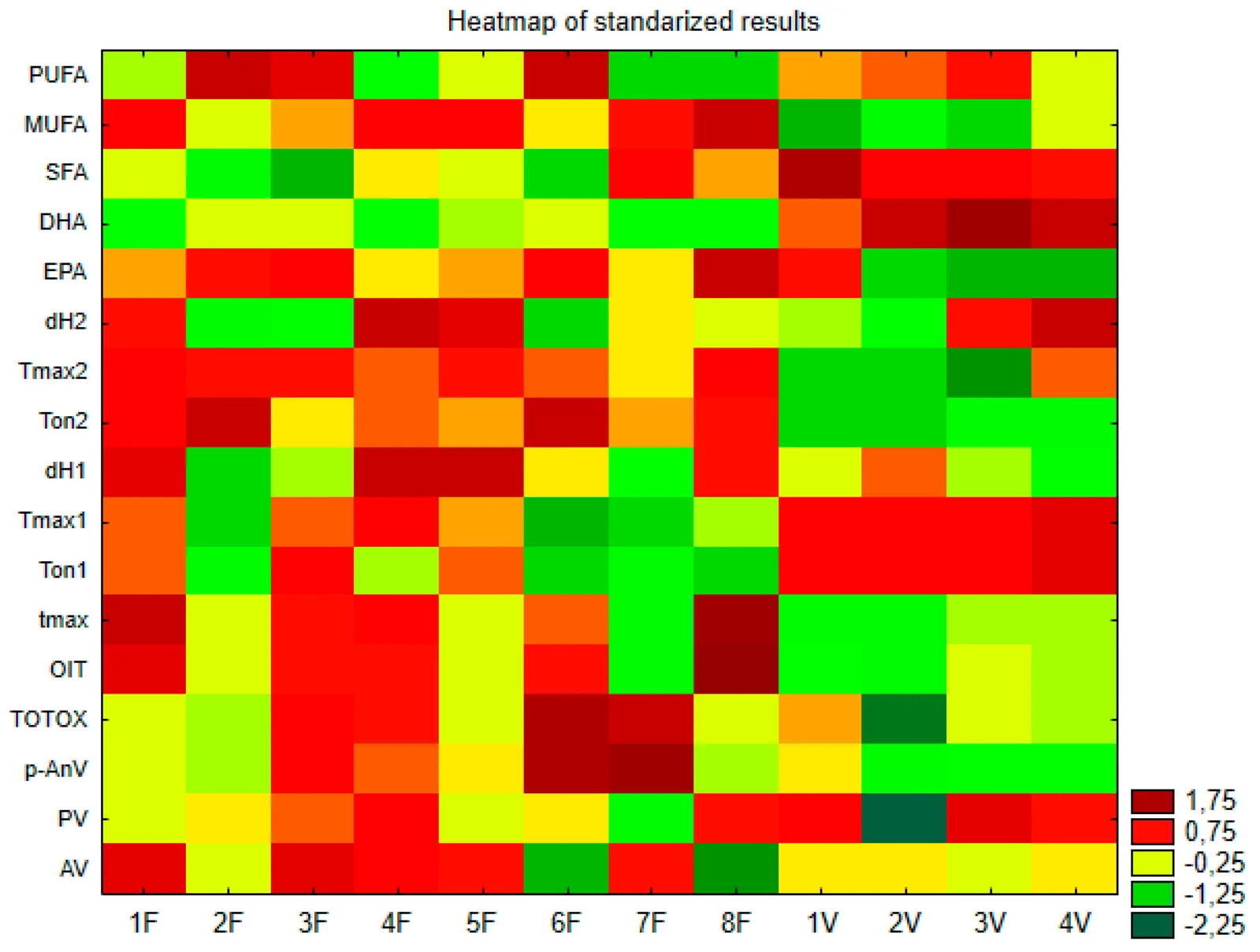
The heatmap of standardized selected results (PUFA—sum of polyunsaturated fatty acids, MUFA—sum of monounsaturated fatty acids, SFA—sum of saturated fatty acids, DHA—percentage share of docosahexaenoic fatty acid, EPA—percentage share of eicosapentaenoic fatty acid, dH—enthalpy recorded in DSC, Ton—onset temperature, Tmax—maximum temperature, tmax—maximum oxidation time, OIT—oxidation induction time, p-AnV—p-anisidine value, PV—peroxide value, AV—acid value) of the omega-3 dietary supplements.

Furthermore, fish oil supplements were separated into two subclusters along PC2, suggesting differences in oxidative status and stability-related parameters within this group. Samples located in the upper-left quadrant (e.g., 2F, 3F, 6F, 7F) can be associated with distinct compositional or oxidation characteristics compared to those in the lower-left quadrant (e.g., 1F, 5F, 8F), indicating differences in the overall characteristics of the analyzed supplements. The heatmap of standardized results (Figure 6) supported this differentiation, highlighting groups of samples with similar oxidative profiles and suggesting a potential influence of formulation factors such as antioxidant systems or packaging.

## 4. Conclusions

The present study demonstrated considerable variability in the quality of omega-3 dietary supplements available on the Polish OTC market, regardless of their origin (fish oil or algae-based). While the fatty acid composition of the analyzed products was generally consistent with their declared source, significant differences were observed in oxidative stability and chemical quality parameters. In particular, variations in peroxide value, anisidine value, and TOTOX index indicated that a proportion of the evaluated supplements may be affected by ongoing or advanced lipid oxidation. The results also confirmed that encapsulation does not fully prevent oxidative degradation and that product stability is influenced by multiple factors, including the source of oil, formulation strategy, antioxidant systems, and packaging conditions. Thermal and oxidative analyses (DSC and PDSC) provided additional insight into the structural and stability-related properties of the lipid fraction, highlighting their usefulness as complementary tools in quality assessment. Importantly, this study demonstrates the applicability of pressure differential scanning calorimetry (PDSC) for evaluating oxidative stability of omega-3 dietary supplements, a relatively underexplored approach in this product category. The combined use of classical chemical indices and advanced analytical techniques offers a more comprehensive evaluation of supplement quality. The obtained findings emphasize the need for continuous monitoring and stricter quality control of omega-3 supplements to ensure their safety, stability, and nutritional value. The present study has some limitations. The relatively small sample size (*n* = 12) and uneven distribution of packaging types between fish- and algae-derived supplements limit the possibility of distinguishing the independent contribution of packaging to the observed differences. Therefore, the findings should be interpreted as an assessment of the analyzed products rather than as representative of the entire Polish market or indicative of long-term storage stability. Nevertheless, this study provides valuable insights into the quality and oxidative stability of commercially available omega-3 supplements and highlights substantial variability among individual products. Furthermore, the application of PDSC alongside conventional chemical quality indices offers a complementary approach to assessing oxidative stability and may contribute to a more comprehensive evaluation of omega-3 supplement quality.

Further research should focus on standardizing analytical approaches and improving formulation strategies to enhance the oxidative stability of these widely consumed products. In addition, studies involving larger sample sets and controlled comparisons of antioxidant formulations and packaging materials are needed to establish evidence-based recommendations for improving the oxidative stability of omega-3 supplements. Additionally, the esterification forms of EPA and DHA (e.g., triglycerides or ethyl esters) and the concentrations of individual antioxidants were not determined in the present study. These factors may influence the physicochemical properties and oxidative stability of omega-3 supplements; therefore, their potential contributions to the observed differences could not be independently assessed.

## Figures and Tables

**Table 1:** The characteristics of the studied omega-3 dietary supplements.

Supplement	Origin	Package	Antioxidant	Coating	Country of Production	Declared EPA%	Declared DHA%
1F	‘Fish’	cardboard	Vitamin E	Beef gelatin	Poland	18	12
2F	‘Fish’	cardboard	Tocopherol mixture	Gelatin	Sweden	33	22
3F	‘Fish’	cardboard	Vitamin E	Gelatin	Poland	33	22
4F	‘Fish’	cardboard	Vitamin E	Gelatin	Poland	18	12
5F	‘Fish’	nontransparent plastic container	α-tocopherol (from sunflower)	Beef gelatin	USA	18	12
6F	‘Fish’	orange glass container	Tocopherol mixture (from soy), rosemary extract	Fish gelatin	Poland	51	34
7F	‘Fish’	orange glass container	Vitamin E	Beef gelatin	Poland	18	12
8F	‘Fish’	orange glass container	Tocopherol mixture	Beef gelatin	Netherlands	16	10
1V	*Schizochytrium* sp.	nontransparent plastic container	Tocopherol mixture, ascorbic acid	Hydroxypropylated starch	Poland	-	62
2V	*Schizochytrium* sp.	nontransparent plastic container	Tocopherol mixture, ascorbic acid	Modified starch	Poland	-	55
3V	*Schizochytrium* sp.	nontransparent plastic container	Tocopherol mixture, ascorbyl palmitate	Tapioca starch	Poland	-	51
4V	*Schizochytrium* sp.; sunflower oil and MCT oil	cardboard	Tocopherol mixture, ascorbic acid	Modified starch	Germany	33	67

**Table 2:** PDSC results for omega-3 dietary supplements.

Sample	OIT (min)	τ_max_ (min)
1F	15.10 ± 0.13 ^d^	18.99 ± 0.23 ^g^
2F	9.84 ± 0.12 ^c^	11.54 ± 0.10 ^cd^
3F	13.86 ± 0.30 ^d^	16.34 ± 0.32 ^ef^
4F	13.39 ± 0.37 ^d^	17.75 ± 0.40 ^fg^
5F	9.52 ± 0.51 ^bc^	12.30 ± 0.48 ^d^
6F	13.45 ± 0.64 ^d^	15.12 ± 0.62 ^e^
7F	7.09 ± 1.80 ^a^	8.95 ± 0.74 ^ab^
8F	18.71 ± 0.19 ^e^	21.49 ± 0.09 ^h^
1V	7.79 ± 0.45 ^ab^	8.77 ± 0.45 ^a^
2V	6.73 ± 0.06 ^a^	8.88 ± 1.12 ^a^
3V	9.35 ± 0.04 ^bc^	10.43 ± 0.09 ^bc^
4V	9.03 ± 0.46 ^bc^	10.79 ± 0.45 ^cd^

OIT—oxidation induction time; τ_max_—maximum oxidation time. Different letters in superscript indicate significantly different results at *p* < 0.05.

**Table 3:** DSC results for omega-3 dietary supplements.

Sample	T_on1_ (°C)	T_max1_ (°C)	ΔH_1_ (J/g)	T_on2_ (°C)	T_max2_ (°C)	ΔH_2_ (J/g)
1F	−57.69 ± 0.03 ^e^	−48.43 ± 0.02 ^e^	14.70 ± 0.09 ^f^	−15.24 ± 0.09 ^e^	−2.88 ± 0.08 ^h^	11.15 ± 0.03 ^f^
2F	−72.56 ± 0.07 ^b^	−67.95 ± 0.08 ^a^	1.35 ± 0.28 ^a^	−11.89 ± 0.58 ^f^	−3.35 ± 0.09 ^gh^	0.88 ± 0.09 ^a^
3F	−53.23 ± 0.05 ^h^	−47.58 ± 0.03 ^f^	5.17 ± 0.15 ^b^	−21.34 ± 2.26 ^b^	−3.62 ± 0.03 ^g^	3.36 ± 0.55 ^bc^
4F	−67.72 ± 0.87 ^c^	−43.12 ± 0.15 ^g^	17.12 ± 1.67 ^g^	−18.35 ± 0.10 ^cd^	−5.74 ± 0.07 ^e^	14.91 ± 0.01 ^g^
5F	−59.53 ± 0.43 ^d^	−49.96 ± 0.03 ^d^	17.08 ± 0.61 ^g^	−19.38 ± 0.51 ^bc^	−3.52 ± 0.03 ^g^	14.16 ± 0.17 ^g^
6F	−74.47 ± 0.18 ^a^	−69.06 ± 0.05 ^b^	9.08 ± 0.51 ^d^	−12.10 ± 1.13 ^f^	−4.89 ± 0.54 ^f^	0.30 ± 0.07 ^a^
7F	−74.22 ± 0.01 ^a^	−67.82 ± 0.01 ^a^	4.06 ± 0.70 ^b^	−19.02 ± 0.85 ^bc^	−8.06 ± 0.18 ^d^	6.75 ± 0.33 ^e^
8F	−74.65 ± 0.41 ^a^	−58.67 ± 0.01 ^c^	12.70 ± 0.64 ^e^	16.26 ± 1.52 ^de^	−2.32 ± 0.16 ^i^	5.54 ± 0.32 ^d^
1V	−54.87 ± 0.16 ^f^	−42.77 ± 0.01 ^h^	7.20 ± 0.31 ^c^	−28.42 ± 0.14 ^a^	−14.56 ± 0.02 ^c^	4.25 ± 0.12 ^c^
2V	−54.37 ± 0.11 ^fg^	−42.27 ± 0.04 ^i^	11.36 ± 0.08 ^e^	−28.69 ± 0.19 ^a^	−15.56 ± 0.14 ^b^	2.88 ± 0.23 ^b^
3V	−53.77 ± 0.21 ^gh^	−43.20 ± 0.08 ^g^	5.54 ± 0.21 ^bc^	−28.04 ± 0.03 ^a^	−17.95 ± 0.01 ^a^	11.37 ± 0.40 ^f^
4V	−51.86 ± 0.04 ^i^	−40.68 ± 0.14 ^j^	4.55 ± 0.12 ^b^	−26.90 ± 0.07 ^a^	−5.47 ± 0.04 ^e^	14.73 ± 0.62 ^g^

T_on1_—onset temperature of the first peak; T_max1_—maximum temperature of the first peak; T_on2_—onset temperature of the second peak; T_max2_—maximum temperature of the second peak; ΔH_1_—enthalpy of the first transition; ΔH_2_—enthalpy of the second transition. Different letters in superscript indicate significantly different results at *p* < 0.05.

**Table 4:** Results of acid value (AV), peroxide value (PV), p-anisidine value (p-AnV), and TOTOX value for omega-3 dietary supplements.

Sample	AV (mg KOH/g)	PV (meq O_2_/kg)	p-AnV	TOTOX
1F	0.82 ± 0.03 ^d^	5.13 ± 0.09 ^d^	13.43 ± 0.07 ^f^	23.69 ± 0.08 ^c^
2F	0.49 ± 0.15 ^b^	5.36 ± 0.05 ^e^	11.67 ± 0.02 ^e^	22.29 ± 0.12 ^b^
3F	0.83 ± 0.01 ^d^	5.84 ± 0.03 ^f^	20.39 ± 0.11 ^i^	32.01 ± 0.10 ^f^
4F	0.73 ± 0.03 ^d^	6.87 ± 0.02 ^i^	17.16 ± 0.02 ^h^	30.84 ± 0.07 ^e^
5F	0.68 ± 0.06 ^cd^	4.98 ± 0.05 ^c^	14.09 ± 0.09 ^g^	23.96 ± 0.10 ^c^
6F	0.20 ± 0.01 ^a^	5.39 ± 0.04 ^e^	25.71 ± 0.07 ^j^	36.43 ± 0.02 ^h^
7F	0.71 ± 0.04 ^d^	3.97 ± 0.08 ^b^	27.03 ± 0.12 ^k^	34.96 ± 0.03 ^g^
8F	0.11 ± 0.01 ^a^	6.34 ± 0.01 ^g^	11.39 ± 0.02 ^d^	24.06 ± 0.02 ^c^
1V	0.53 ± 0.07 ^bc^	6.68 ± 0.02 ^h^	14.21 ± 0.01 ^g^	27.51 ± 0.10 ^d^
2V	0.54 ± 0.03 ^bc^	2.08 ± 0.03 ^a^	7.46 ± 0.02 ^a^	11.57 ± 0.11 ^a^
3V	0.45 ± 0.06 ^b^	6.97 ± 0.02 ^j^	9.88 ± 0.10 ^b^	23.79 ± 0.04 ^c^
4V	0.54 ± 0.03 ^bc^	6.25 ± 0.04 ^g^	10.26 ± 0.05 ^c^	22.70 ± 0.11 ^b^

Different letters in superscript indicate significantly different results at *p* < 0.05.

## Data Availability

The original contributions presented in this study are included in the article/Supplementary Material. Further inquiries can be directed to the corresponding authors.

## Supplementary Materials

The following supporting information can be downloaded at: https://www.mdpi.com/article/10.3390/foods15213786/s1, Table S1: GC results for omega-3 dietary supplements.
